# Intramyocardial Left Anterior Descending Artery Extending Toward the Right Ventricle Demonstrated on Coronary CT Angiography

**DOI:** 10.3390/diagnostics16081116

**Published:** 2026-04-08

**Authors:** Mira Yuniarti, Jonggi Mathias Tamba, Gilbert Sterling Octavius

**Affiliations:** Department of Radiology, Faculty of Medicine, Universitas Pelita Harapan, Tangerang 36136, Indonesia

**Keywords:** intramyocardial coronary artery, myocardial bridging, coronary CT angiography, left anterior descending artery, coronary anomalous variant

## Abstract

Intramyocardial coronary artery course is a rare anatomical variant that can be increasingly recognized with coronary computed tomography angiography (CCTA). We present the case of a 22-year-old male who underwent CCTA for evaluation of chest pain. Imaging demonstrated an unusual course of the left anterior descending artery (LAD), which traversed toward the right ventricular cavity over an approximately 21 mm segment. Multiplanar reconstructions and three-dimensional volume-rendered images clearly depicted the intramyocardial trajectory of the vessel. Although usually asymptomatic, recognition of this variant is important because intramyocardial coronary arteries may be vulnerable to injury during intracardiac procedures. This case highlights the role of CCTA in accurately characterizing a rare intracavitary LAD course with clear delineation of its intramyocardial-to-intracavitary trajectory toward the right ventricle using multiplanar and three-dimensional reconstructions.

**Figure 1:**
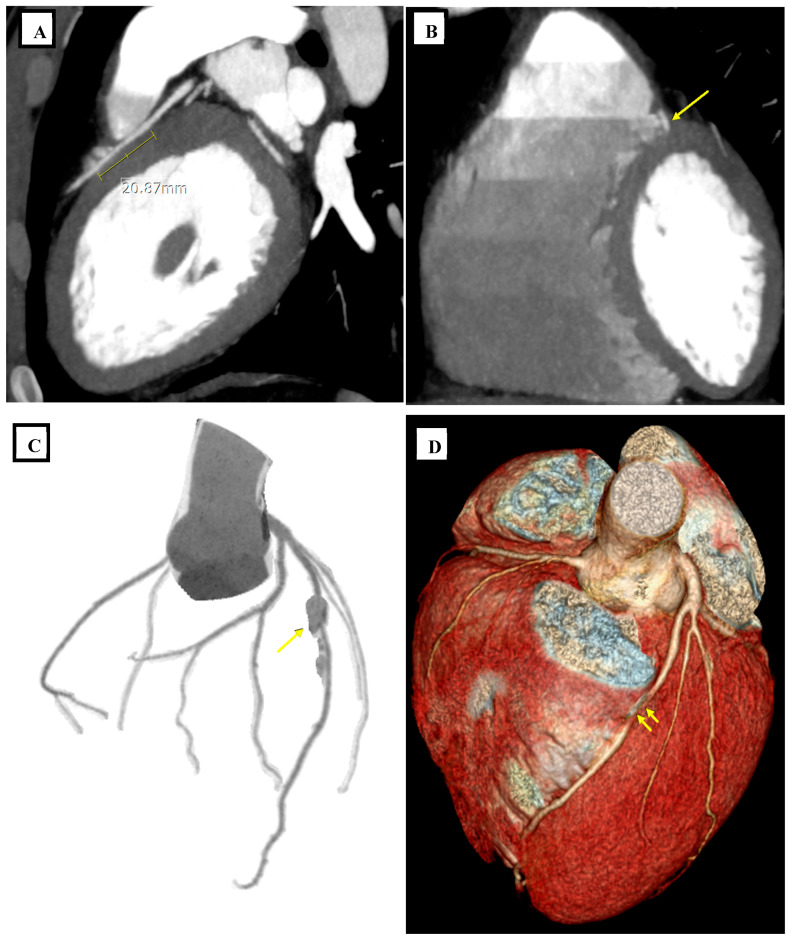
A 22-year-old male presenting with chest pain underwent coronary computed tomography angiography for further evaluation. Coronary computed tomography angiography (CCTA) was performed using contrast enhancement (400 mg iodine/mL, 80 mL at 5 mL/s) with retrospective ECG-gated acquisition. The patient had a baseline heart rate of 42 beats per minute, and no pharmacological heart rate control was required. Images were reconstructed with a slice thickness of approximately 0.6–0.75 mm and evaluated using multiplanar and three-dimensional reconstructions. (**A**) Reformatted sagittal view demonstrates a myocardial bridge of the left anterior descending artery (LAD) extending toward the right ventricle (RV), with an estimated length of approximately 21 mm and an intramyocardial depth of approximately 1.8 mm. (**B**) Reformatted coronal view confirms the intramyocardial course of the LAD (arrow). (**C**) Curved planar reformation demonstrates no focal luminal narrowing at the tunneled segment (arrow). (**D**) Volume-rendered reconstruction delineates the intramyocardial course of the LAD traversing toward the RV cavity (arrows). No significant luminal narrowing or features of hemodynamic compromise were identified, and the anatomical finding was considered incidental in relation to the patient’s presenting symptoms. Coronary arteries typically follow a subepicardial course along the surface of the heart. However, segments of a coronary artery may descend intramurally within the myocardium and become covered by myocardial fibers, a phenomenon referred to as myocardial bridging (MB). In this configuration, the intramural arterial segment is termed the “tunneled artery,” while the overlying myocardial fibers form the myocardial bridge. A large, multi-detector CT study conducted in an Indonesian population reported a prevalence of 44.3%, with the left anterior descending artery (LAD) involved in nearly all cases (99.6%), highlighting the LAD as the most frequently affected vessel [1]. This case illustrates an uncommon intramyocardial LAD course toward the right ventricle detected using coronary CT angiography. It is important to distinguish myocardial bridging from an intracavitary coronary course. In myocardial bridging, the coronary artery remains embedded within the myocardium and is covered by myocardial fibers, whereas in an intracavitary course, the vessel traverses within a cardiac chamber before returning to the epicardial surface [2]. This distinction is clinically relevant because intracavitary segments may be more directly exposed to intracardiac instrumentation, whereas myocardial bridging is primarily associated with dynamic systolic compression and related hemodynamic effects [3]. While myocardial bridging is relatively common, intramyocardial coronary artery courses represent a distinctly rare anatomical variant. In this condition, a segment of the coronary artery traverses within a cardiac chamber before returning to the epicardial surface. The reported prevalence ranges from 0.054% to 0.36%, and the condition is increasingly recognized with the wider use of coronary CT angiography [4]. A large CCTA analysis of 7847 patients identified intracavitary coronary arteries in 0.36% of cases, with the LAD coursing through the right ventricle in approximately 0.14% of patients, emphasizing the rarity of this anatomical configuration [5]. Similarly, a retrospective coronary CTA study evaluating more than 31,000 examinations identified only 17 cases of intra-right ventricular coronary course, all involving the LAD. In these patients, the intraventricular segment most frequently involved the mid or distal LAD and typically followed a trajectory along the border between the right ventricular free wall and the interventricular septum, often within trabeculae carneae. The mean intraventricular segment length reported in that series was approximately 25 mm, comparable to previously described myocardial bridge lengths [6]. Although myocardial bridging is generally considered a benign anatomical variant, its clinical implications remain debated. The tunneled coronary segment may demonstrate dynamic systolic compression, often described angiographically as a “milking effect [7]”. In most patients, this phenomenon is asymptomatic; however, myocardial bridging has been associated in some cases with myocardial ischemia, arrhythmias, and angina-like symptoms. Hemodynamic alterations caused by myocardial bridging may also promote atherosclerotic plaque formation proximal to the bridged segment, while the tunneled portion tends to remain relatively protected from atherosclerosis [1]. The relatively shallow intramyocardial depth (1.8 mm) observed in this case may partly explain the absence of luminal narrowing or apparent hemodynamic compromise on CCTA. Coronary CT angiography plays a central role in the detection of such anomalies because it allows high-resolution three-dimensional visualization of the coronary artery course relative to cardiac chambers and myocardial structures [6]. Unlike conventional coronary angiography, which provides limited spatial information, CCTA enables accurate depiction of intramyocardial and intracavitary coronary segments and therefore facilitates the recognition of rare anatomical variants. In summary, while myocardial bridging most commonly involves the LAD and is frequently detected on coronary CT angiography, this case demonstrates a rare intracavitary LAD course with clear visualization of its transition from an intramyocardial to an intracavitary trajectory toward the right ventricle. This detailed anatomical depiction using CCTA adds to the existing literature by emphasizing the capability of advanced imaging reconstructions to precisely characterize such uncommon variants.

## Data Availability

The raw data supporting the conclusions of this article will be made available by the authors on request.

## Abbreviations

The following abbreviations are used in this manuscript:

LAD	Left anterior descending
CCTA	Coronary computed tomography angiography
RV	Right ventricle

## References

[1] Koesbandono, Lukito A.A., Muljadi R., Yuniarti M., Sindunata N.A., Sarikie A., Pratama T.A., Thio R.S., Christanti J., Octavius G.S. (2024). High Prevalence of Myocardial Bridging Detected in an Indonesian Population Using Multi-Detector Computed Tomography. Medicina.

[2] Gać P., Siudek B., Głuszczyk A., Plizga J., Grajnert F., Poręba R. (2024). Computed Tomography Angiography as a Method for Diagnosing Intracavitary Coronary Arteries. Diagnostics.

[3] Seo C.-O., Kim H., Koh J.-S. (2025). Fractional Flow Reserve in the Left Anterior Descending Artery. J. Clin. Med..

[4] Hussein H., Elshall A., Youssef A., Hekal S., Shaaban M. (2023). Combined intra-cavitary course of left anterior descending artery and myocardial bridge of right coronary artery in right ventricle hypertrophy: A case report. Eur. Heart J. Case Rep..

[5] Buckley C.M., Rosamond T., Hegde S.R., Wetzel L. (2017). The Intracavitary Coronary Artery: A Rare Anomaly with Implications for Invasive Cardiac Procedures—Demonstration by Coronary Computed Tomography Angiography. J. Am. Coll. Cardiol..

[6] Tyczyński P., Skowroński J., Opolski M.P., Pręgowski J., Kępka C., Kruk M., Orczykowski M., Łazarczyk H.E., Witkowski A., Michałowska I. (2020). Intra-Right Ventricle Course of the Coronary Arteries on Computed Tomography Angiography. J. Comput. Assist. Tomogr..

[7] Gurewitch J., Gotsman M.S., Rozenman Y. (1999). Right ventricular myocardial bridge in a patient with pulmonary hypertension—A case report. Angiology.

